# The associations of dietary manganese, iron, copper, zinc, selenium and magnesium with cognitive outcomes in Chinese adults: a cross sectional study in Shanghai

**DOI:** 10.3389/fnut.2024.1424614

**Published:** 2024-12-04

**Authors:** Yujie Chen, Zhuo Sun, Yali Zhang, Ren Zhou, Xingyu Lin, Yuewen Du, Jiayi Xu, Qi Xu, Jiajie Zang

**Affiliations:** ^1^School of Public Health, Shanghai University of Traditional Chinese Medicine, Shanghai, China; ^2^Division of Health Risk Factors Monitoring and Control, Shanghai Municipal Center for Disease Control and Prevention, Shanghai, China; ^3^Department of Anesthesiology, The Ninth People's Hospital of Shanghai, Jiao Tong University School of Medicine, Shanghai, China

**Keywords:** dietary minerals, low MMSE scores, cognitive function, dietary copper, dietary magnesium

## Abstract

**Introduction:**

The role of individual nutrients including vitamins and minerals in cognitive function gained increasing attention in recent years. With regard to the association between dietary minerals and cognitive function, the results of human studies are inconclusive. The objective of this study was to explore the association between mineral intake and cognitive function using the data from Shanghai Health and Nutrition Survey (SHNS) in 2018.

**Methods:**

In total, 835 adults were included in a crosssectional study, and completed a three-day dietary record to estimate their average daily intake of minerals. Mini-Mental State Examination (MMSE) was used for the assessment of cognitive function, and logistic regression analyses were performed on participants to examine the association between dietary mineral intake and cognitive performance. The participants were divided into tertiles according to their mineral intake.

**Results:**

Participants in the second and third tertile of the dietary copper intake had lower rates of low MMSE scores compared to those in the lowest tertile. We found the adjusted OR and 95%CI values were 0.44 (0.21–0.89) and 0.40 (0.16–0.94), respectively. Participants in the second tertile of dietary magnesium intake showed a trend of lower rates of low MMSE score compared to those in the lowest tertile (*p* = 0.06). The adjusted OR and 95%CI values were 0.35 (0.16–0.72). No significant association were observed between any of the other minerals including iron, zinc, selenium and manganese and cognitive function.

**Discussion:**

Our findings suggest that dietary intake of copper and magnesium may have a protective effect on cognitive performance in elderly over 60 years old. To prevent cognitive decline, elderly should get recommended amounts of copper and magnesium from diet or supplements.

## Introduction

With the elderly population increasing dramatically, cognitive impairment has become a public health challenge. It is estimated that around 55 million people suffer from dementia globally and about 10 million new cases are identified yearly (1). Moreover, its global prevalence is projected to increase to 132 million by 2050 (2). Mild cognitive impairment (MCI), characterized by a decline in cognitive function that occurs with typical aging, is the early stage of cognitive impairment between the aging and dementia (3). Since there is no effective treatment to slow down or reverse the dementia (4), delaying the transition from mild cognitive impairment to dementia is imminent.

In recent decades, there is growing evidence supporting the key role of diet and nutrition in the onset and severity of age-related cognitive deterioration (5). Among these, minerals have gained great attention. On the one hand, minerals, such as iron, manganese, copper, zinc, and magnesium serve as essential components of thousands of enzymes and proteins, involving DNA repair and brain development (6). They also play an important role in regulating cellular function and nerve transmission, which can thus decelerate or prevent the process of cognitive decline (7). On the other hands, it is well established that amyloid plaques contain high concentrations of copper, iron, and zinc, indicating the crucial role of these trace elements in the pathogenesis of neurological diseases such as Alzheimer’s disease (AD).

Human studies with regard to the association between dietary intake of minerals and cognitive performance are also controversial and limited. For example, a cross-sectional data analysis from National Health and Nutrition Examination Surveys 2011–2014 (NHANES 2011–2014) suggested the copper intake below the inflection point was positively and independently associated with cognitive function (8). Another study from 10,269 participants of the Atherosclerosis Risks in Communities Study found that high dietary copper intake combined with a high intake of saturated fat may increase the risk of cognitive impairment (9). A 15-year longitudinal study suggested that higher iron intake is associated with poorer cognition in older Chinese individuals (10). However, the study from NHANES 2011–2014 found that raised iron levels in the serum were linked to a decreased risk of cognitive impairment (11). Moreover, Shanghai is the city with the highest level of aging in China. This study aimed to examine the relationship between dietary minerals including manganese, iron, copper, zinc, selenium and magnesium and cognition performance tested by Mini-Mental State Examination (MMSE) an well-known instrument for cognitive function, using the data from Shanghai Health and Nutrition Survey (SHNS) in 2018. We hope our study will help provide evidence for nutritional intervention to prevent or delay age related cognitive decline and maintain a good quality of life among elderly adults.

## Methods

### Study design and participants

The current study was a cross sectional analysis based on data from Shanghai Health and Nutrition Survey (SHNS) in the cycle of 2018 with a total of 1,516 participants. SHNS was carried out by the Shanghai Municipal Center for Disease Control and Prevention every 3 years. It is designed to examine the effects of the health, nutrition, and family planning policies and programs implemented by national and local governments. The study was approved by the Ethical Review Committee of the Shanghai Municipal Centers for Disease Control and Prevention. The written informed consents were obtained from all participants enrolled in the study.

**Figure 1:**
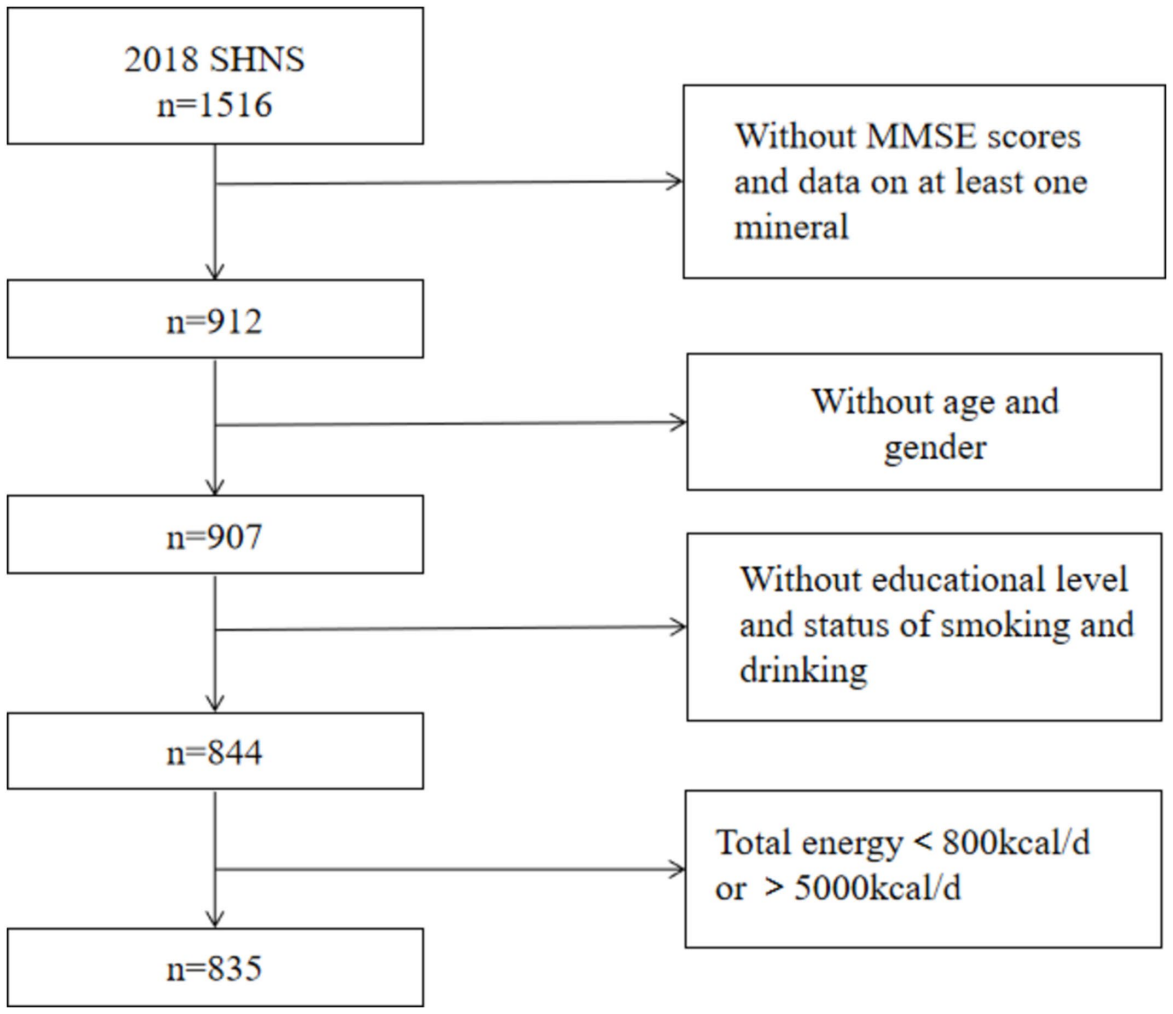
Flow chart of the selection process.

Our analyses were limited to participants who completed the MMSE scale and had recorded intake of at least one mineral (*n* = 912). Those who did not complete the general questionnaire or had missing information on age, gender, educational level, smoking status or drinking status were excluded (*n* = 68). In addition, participants with extreme values of total dietary energy intake (>5,000 kcal/d or <800 kcal/d) were also excluded (*n* = 9). Finally, 835 participants (342 individuals less than 60 years old, 493 individuals aged over 60 years old were included in the analyses). The flow chart of participants was shown in Figure 1.

### Dietary assessment

Dietary intake was obtained from the participants for three consecutive days on a 24-h recall basis during 2018. Individuals were asked about the exact type and weight of foods they consumed in 3 days, including 2 weekdays and 1 weekend day. The field interviewers had received three days of training about the collection of dietary data and had prior experience in both nutrition work and national surveys. The intake of each food group (not including dietary supplements) was converted from categorical responses to numeric variables, and the dietary intake of energy and certain nutrients were calculated using Chinese food composition table containing detailed lists of food types and nutrient values for all kinds of foods. We also calculated the contribution of various foods to copper and magnesium intake based on dietary assessment. The food categories include grains, vegetables, legumes, sea foods, meats and poultry, fruits, cookies and snacks, nuts, eggs, fungi and algae.

### Cognitive performance outcomes

The Mini-Mental State Examination (MMSE) was used for assessing cognition in all participants. The participants were face-to-face interviewed for 30 min to evaluate cognitive function through five domains: orientation (10 points), recall (3 points), registration (3 points), attention/computation (5 points), and language (9 points). MMSE score ranges from 0 to 30. A higher MMSE score indicates the better cognitive function. In addition, the threshold for the MMSE score was set at 27, with a score of less than 27 considered as low MMSE score at greater risk of being diagnosed with dementia (12).

### Covariates

To control for the effect of confounders on the results, the statistical model was adjusted for covariates to minimize the potential confounding bias. Age, gender, educational level were obtained from in-person household interviews. Educational level was classified into three categories: less than high school, high school (including secondary technical school and vocational school), higher than high school. Total energy intake was obtained from the 3 consecutive 24-h recalls. Smoking status was divided into two groups (never smoked, smoked) based on the self-report of the participants to the question “Ever smoked cigarettes?.” Participants were defined as alcohol drinkers if they had ever drank beer/alcohol in the previous year. Chinese version of the 14-item Perceived Stress Scale (PSS-14) was used to assess levels, and the threshold for the PSS-14 score was set at 28, with a score of more than 28 considered to have some sense of stress.

### Statistical analysis

Medians and percentiles was used for describing the characteristics of all variables between the different groups. Dietary manganese, iron, copper, zinc and magnesium intakes were categorized into low moderate and high intake tertiles for each subject. Logistic regression analyses were conducted to examine the associations between manganese, iron, copper, zinc and magnesium intakes and the level of MMSE scores. The model 1 was adjusted for age (years), gender and total daily energy intake (kcal/d). Model 2 was further adjusted for educational level (less than high school, high school, higher than high school), smoking status (yes, or no) and alcohol drinking (yes, or no), and model 3 was built on model 2 plus the level of perceived stress score. Stratified analyses by two age groups were also conducted to further address the concern on the confounding by age. All statistical analyses were performed with R (4.0.5) and *p* < 0.05 was considered statistically significant.

## Results

### Characteristics of the participants

Characteristics of the participants by different age groups were summarized in Table 1. A total of 835 participants were included in the study, among whom 49.3% were male and 20.4% had education beyond high school. The percentage of smokers and drinkers was 75.0 and 79.3%, respectively. The median age of participants was 62 years old. The median total dietary energy intake was 1865 kcal and the median level of dietary mineral intake was 4.62 [3.45, 5.99] mg/d for manganese, 18.25 [14.12, 24.63] mg/d for iron, 1.51 [1.13, 2.04] mg/d for copper, 10.14 [8.26, 12.77] mg/d for zinc, 49.61 [38.32, 63.54] μg/d for selenium and 256.88 [198.13, 333.99] mg/d for magnesium. Among all participants, there were 73 of them with low MMSE (score less than 27), accounting for 8.7% of the total.

**Table 1:** Characteristics of the participants by age groups.

Characteristic	Total	Age < 60y	Age ≥ 60y	*p*
*N*	835	342	493	
Age in years at screening, median	62.00 [55.00, 68.00]	52.00 [46.00, 56.00]	67.00 [63.00, 71.00]	<0.001
**Gender, *n* (%)**				
Male	412 (49.3)	165 (48.2)	247 (50.1)	0.623
Female	423 (50.7)	177 (51.8)	246 (49.9)	
**Education, *n* (%)**				
<High school	310 (37.1)	91 (26.6)	219 (44.4)	<0.001
High school	355 (42.5)	153 (44.7)	202 (41.0)	
>High school	170 (20.4)	98 (28.7)	72 (14.6)	
**Smoking, *n* (%)**				
Yes	626 (75.0)	254 (74.3)	372 (75.5)	0.745
No	209 (25.0)	88 (25.7)	121 (24.5)	
**Drinking, *n* (%)**				
Yes	662 (79.3)	265 (77.5)	397 (80.5)	0.298
No	173 (20.7)	77 (22.5)	96 (19.5)	
Total energy (kcal), median [IQR]	1864.54 [1565.92, 2248.22]	1990.35 [1615.85, 2334.14]	1781.07 [1533.02, 2164.08]	<0.001
Dietary manganese (mg/d), median [IQR]	4.62 [3.45, 5.99]	4.68 [3.23, 6.13]	4.56 [3.51, 5.96]	0.897
Dietary iron (mg/d), median [IQR]	18.25 [14.12, 24.63]	19.18 [14.52, 24.91]	17.67 [13.95, 23.98]	0.119
Dietary copper (mg/d), median [IQR]	1.51 [1.13, 2.04]	1.52 [1.12, 2.09]	1.51 [1.13, 2.02]	0.790
Dietary zinc (mg/d), median [IQR]	10.14 [8.26, 12.77]	10.60 [8.81, 13.41]	9.91 [8.04, 12.18]	0.001
Dietary selenium (μg/d), median [IQR]	49.61 [38.32, 63.54]	50.91 [39.58, 66.69]	48.84 [37.38, 62.22]	0.028
Dietary magnesium (mg/d), median [IQR]	256.88 [198.13, 333.99]	263.19 [196.65, 337.83]	251.57 [198.87, 328.12]	0.612
Low MMSE score, *n* (%)	73 (8.7)	11 (3.2)	62 (12.6)	<0.001

According to the median age of 62 years, compared with the latest (2023) Dietary Reference Intakes (DRIs) for Chinese residents, the median total dietary energy intake basically met the required estimated energy requirement (EER), and the median dietary mineral intake was higher than the recommended intake (RNI) for manganese (4 mg/d), iron (12 mg/d for males, and 10 mg/d for females) and copper (0.8 mg/d). While zinc intake met the RNI (12 mg/d for males and 8.5 mg/d for females) while the selenium intake failed to meet the dietary requirement (60 μg/d).

Participants over the age of 60 had a lower percentage of tertiary education and a higher percentage of low MMSE, smoking and drinking compared to participants under the age of 60. In addition, participants over 60 years of age had lower intakes of total dietary energy and each of the dietary minerals than those under 60 years of age. There were significant differences between people those under 60 and those over 60 years old in the distribution of education, MMSE score, total energy intake (*p* < 0.001), dietary selenium and zinc intake (*p* < 0.05). People over the age of 60 had lower education level, lower MMSE score, less total energy intake, and less dietary zinc and selenium intake than those under the age of 60.

Characteristics of the participants by cognitive performance were summarized in Table 2. Among participants under 60 years of age, there were no significant differences across all characteristics. Among the group over 60 years old, participants with low MMSE were older (*p* < 0.001) and had higher rates of alcohol drinking (*p* < 0.05), lower education level (*p* < 0.05), less total energy intake (*p* < 0.05), less copper intake (*p* < 0.001) and tended to have less magnesium intake (*p* = 0.054).

**Table 2:** Characteristics of the participants by cognitive performance in different age groups.

Characteristic	Age < 60y				Age ≥ 60y			
	Total	Normal MMSE	Low MMSE	*p*	Total	Normal	Low MMSE	*p*
*N*	342	331	11		493	431	62	
Age in years at screening, median [IQR]	52.00 [46.00, 56.00]	52.00 [46.00, 56.00]	55.00 [53.00, 56.00]	0.247	67.00 [63.00, 71.00]	66.00 [63.00, 70.00]	70.00 [67.00, 77.00]	<0.001
**Gender, *n* (%)**								
Male	165 (48.2)	160 (48.3)	5 (45.5)	1.000	247 (50.1)	218 (50.6)	29 (46.8)	0.590
Female	177 (51.8)	171 (51.7)	6 (54.5)		246 (49.9)	213 (49.4)	33 (53.2)	
**Education, *n* (%)**								
<High school	91 (26.6)	85 (25.7)	6 (54.5)	0.105	219 (44.4)	185 (42.9)	34 (54.8)	0.010
High school	153 (44.7)	149 (45.0)	4 (36.4)		202 (41.0)	187 (43.4)	15 (24.2)	
>High school	98 (28.7)	97 (29.3)	1 (9.1)		72 (14.6)	59 (13.7)	13 (21.0)	
**Smoking, *n* (%)**								
Yes	254 (74.3)	247 (74.6)	7 (63.6)	0.483	372 (75.5)	321 (74.5)	51 (82.3)	0.209
No	88 (25.7)	84 (25.4)	4 (36.4)		121 (24.5)	110 (25.5)	11 (17.7)	
**Drinking, *n* (%)**								
Yes	265 (77.5)	256 (77.3)	9 (81.8)	1.000	397 (80.5)	341 (79.1)	56 (90.3)	0.039
No	77 (22.5)	75 (22.7)	2 (18.2)		96 (19.5)	90 (20.9)	6 (9.7)	
Total energy (kcal), median [IQR]	1990.35 [1615.85, 2334.14]	1983.59 [1607.23, 2343.77]	2218.05 [1895.64, 2284.55]	0.280	1781.07 [1533.02, 2164.08]	1797.74 [1563.36, 2162.76]	1716.83 [1358.56, 2182.11]	0.067
Dietary manganese (mg/d), median [IQR]	4.68 [3.23, 6.13]	4.70 [3.24, 6.13]	4.14 [3.42, 5.73]	0.937	4.56 [3.51, 5.96]	4.64 [3.55, 5.97]	4.14 [3.35, 5.46]	0.159
Dietary iron (mg/d), median [IQR]	19.18 [14.52, 24.91]	19.22 [14.63, 24.91]	16.42 [13.39, 23.67]	0.650	17.67 [13.95, 23.98]	17.80 [14.10, 23.98]	16.84 [13.23, 23.26]	0.462
Dietary copper (mg/d), median [IQR]	1.52 [1.12, 2.09]	1.51 [1.12, 2.09]	1.85 [1.04, 2.17]	0.644	1.51 [1.13, 2.02]	1.56 [1.16, 2.04]	1.23 [0.98, 1.67]	0.001
Dietary zinc (mg/d), median [IQR]	10.60 [8.81, 13.41]	10.62 [8.82, 13.42]	10.41 [7.84, 12.54]	0.746	9.91 [8.04, 12.18]	9.91 [8.09, 12.36]	9.61 [7.36, 11.79]	0.208
Dietary selenium (μg/d), median [IQR]	50.91 [39.58, 66.69]	50.83 [39.55, 66.53]	61.73 [46.14, 69.94]	0.187	48.84 [37.38, 62.22]	49.09 [38.29, 62.66]	47.13 [32.59, 59.04]	0.115
Dietary magnesium (mg/d), median [IQR]	263.19 [196.65, 337.83]	263.19 [196.75, 338.57]	263.19 [196.07, 288.83]	0.640	251.57 [198.87, 328.12]	256.88 [201.15, 332.47]	220.19 [192.59, 301.01]	0.054

### The association between mineral intake and cognitive function

Logistic regression analyses were performed on participants over 60 years of age to examine the association between dietary intake of manganese, iron, copper, zinc, and magnesium and the prevalence of low cognitive performance, with the lowest tertile of intake as the referent category. The associations between different dietary mineral intake and low MMSE of participants over 60 years of age were presented in Table 3. The OR with 95% CIs of model 1 indicated that dietary copper intake and magnesium intake had significant inverse associations with low MMSE score (*p* < 0.05). After adjustment for other potential confounders, participants in the second (≥1.24 to<1.78 mg/d) and third tertile (≥1.78 mg/d) of the dietary copper intake had lower rates of low MMSE scores compared to those in the lowest tertile (*p* < 0.05). The adjusted OR and 95%CI values were 0.44 (0.21–0.89) and 0.40 (0.16–0.94), respectively. In addition, participants in the second tertile of dietary magnesium (≥217.86 to<297.56 mg/d) intake showed a trend of lower rates of low MMSE score compared to those in the lowest tertile (*p* = 0.06). The adjusted OR and 95%CI values were 0.35 (0.16–0.72). Additionally, further adjustment for iron as a possible confounder of MMSE scores did not have an effect on the results (Supplementary Table S1).

**Table 3:** Associations between dietary mineral intakes and low MMSE in participants over 60 years old.

	Model 1		Model 2		Model 3	
Dietary manganese (mg/d)	OR (95%CI)	*p* trend	OR (95%CI)	*p* trend	OR (95%CI)	*p* trend
T1 (<3.82)	Reference	0.069	Reference	0.072	Reference	0.097
T2 (≥3.82 to <5.25)	0.58 (0.28–1.16)		0.59 (0.29–1.18)		0.57 (0.28–1.17)	
T3 (≥5.25)	0.46 (0.19–1.07)		0.46 (0.19–1.08)		0.49 (0.20–1.16)	
Liner	0.79 (0.32–1.90)		0.83 (0.33–2.02)		0.88 (0.35–2.16)	
**Dietary iron (mg/d)**						
T1 (<14.99)	Reference	0.540	Reference	0.597	Reference	0.621
T2 (≥14.99 to <21.13)	0.83 (0.41–1.69)		0.85 (0.42–1.74)		0.85 (0.41–1.73)	
T3 (≥21.13)	0.77 (0.33–1.78)		0.80 (0.34–1.85)		0.81 (0.34–1.89)	
Liner	0.74 (0.29–1.79)		0.79 (0.30–1.95)		0.82 (0.31–2.04)	
**Dietary copper (mg/d)**						
T1 (<1.24)	Reference	0.027	Reference	0.030	Reference	0.028
T2 (≥1.24 to <1.78)	0.44 (0.21–0.88)		0.43 (0.21–0.88)		0.44 (0.21–0.89)	
T3 (≥1.78)	0.40 (0.17–0.93)		0.41 (0.17–0.95)		0.40 (0.16–0.94)	
Liner	0.45 (0.18–1.07)		0.46 (0.18–1.11)		0.46 (0.18–1.12)	
**Dietary zinc (mg/d)**						
T1 (<8.68)	Reference	0.691	Reference	0.643	Reference	0.641
T2 (≥8.68 to <11.24)	0.74 (0.35–1.55)		0.76 (0.36–1.60)		0.80 (0.37–1.70)	
T3 (≥11.24)	1.25 (0.51–3.08)		1.29 (0.52–3.20)		1.28 (0.51–3.17)	
Liner	1.16 (0.26–5.29)		1.34 (0.29–6.15)		1.29 (0.29–5.87)	
**Dietary selenium (μg/d)**						
T1 (<41.54)	Reference	0.872	Reference	0.951	Reference	0.957
T2 (≥41.54 to <56.65)	0.54 (0.26–1.12)		0.56 (0.26–1.17)		0.57 (0.27–1.21)	
T3 (≥56.65)	0.96 (0.45–2.03)		1.04 (0.47–2.28)		1.04 (0.47–2.28)	
Liner	0.55 (0.25–1.19)		0.6 (0.27–1.33)		0.59 (0.19–1.31)	
**Dietary magnesium (mg/d)**						
T1 (<217.86)	Reference	0.045	Reference	0.060	Reference	0.061
T2 (≥217.86 to <297.56)	0.37 (0.17–0.76)		0.36 (0.17–0.75)		0.35 (0.16–0.72)	
T3 (≥297.56)	0.45 (0.19–1.03)		0.48 (0.20–1.11)		0.48 (0.20–1.12)	
Liner	0.49 (0.17–1.41)		0.56 (0.18–1.67)		0.58 (0.19–1.73)	

**Figure 2:**
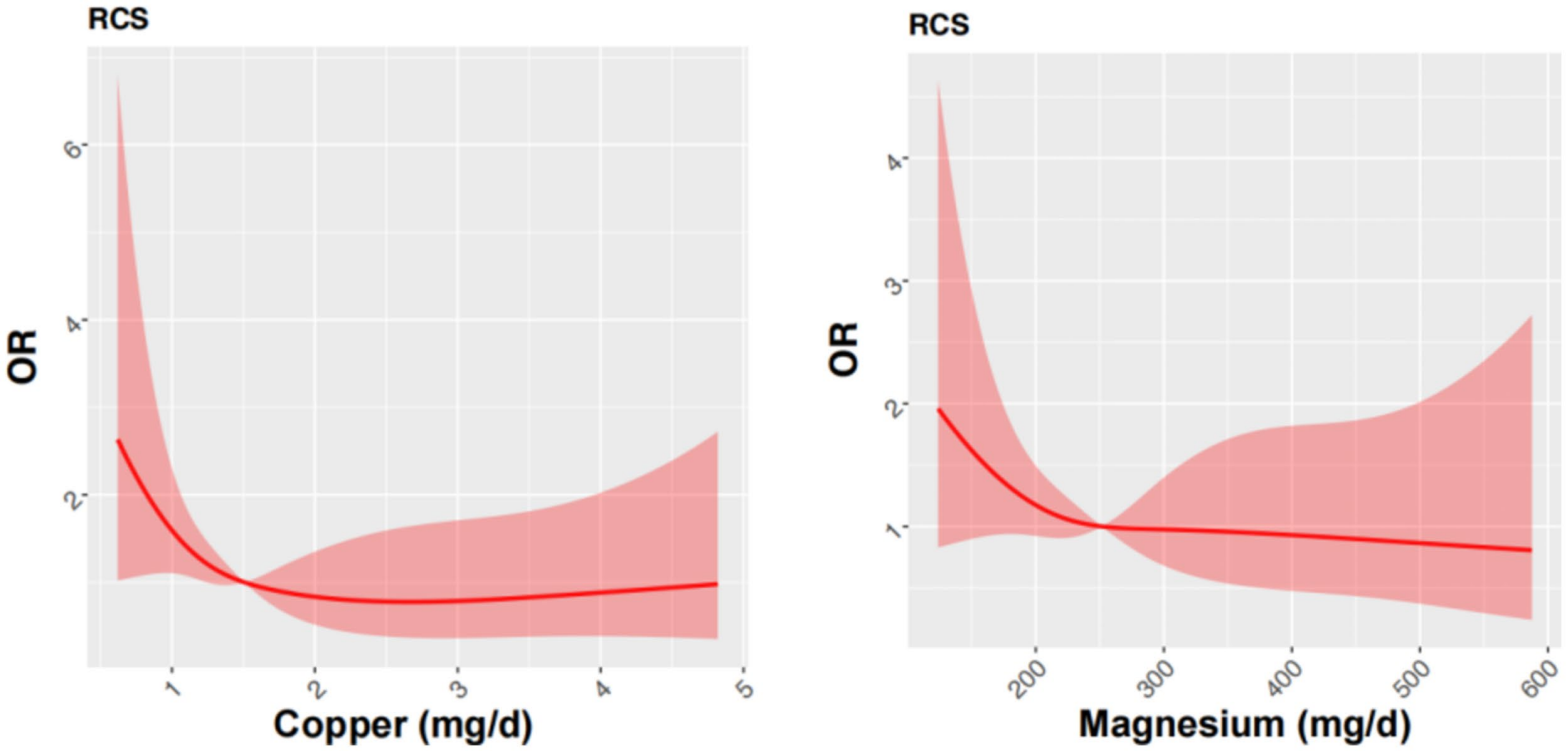
Restricted cubic spline models for the relationship between dietary copper and magnesium intake and the risk of low MMSE after matching. Restricted cubic spline model of the ORs of low cognitive performance with dietary copper and magnesium intakes. The model was adjusted for sex, age, educational level, smoking and alcohol intake. The 95% CIs of the adjusted ORs were represented by the red-shaded area. OR, odds ratio.

To further elucidate the relationship between dietary copper and magnesium intake and cognitive performance, we performed an restricted cubic spline (RCS) analysis (Figure 2). We found the prevalence of low cognitive performance in MMSE decreased with increasing intakes of dietary magnesium intake, and showed a nonlinear L-shaped relationship.

Since our results suggested that dietary intake of copper and magnesium may have a protective effect on cognitive performance in elderly over 60 years old, we further calculated the contribution of various foods to copper and magnesium intake. The results showed that grains were the primary contributor to the copper and magnesium intake (32%), followed by vegetables (11.9%), legumes (10.7%), sea food (10.4%), meats and poultry (8.5%), fruits (5%), fast food (4.3%), cookies and snacks (3.3%), eggs (2.6%), fungi and alage (2.4%), and nuts (2.2%) (the data was shown in Supplementary Figure S1).

## Discussion

With the increased aging population, cognitive impairment has become a global public health problem. A recent meta-analysis based on 96 studies reported an overall dementia prevalence of 5.3% for Chinese population over 60 years old (13). The overall prevalence of MCI in the Chinese population over 60 is estimated to be 15.5%, with 11.1% in those aged 60–69 years, and the prevalence increases with age (14, 15). Since cognitive impairment imposes a heavy burden on the public and health care systems, it is necessary to discover the influencing factors of cognitive dysfunction and take early intervention to prevent the development from MCI to dementia.

Diet is a modifiable lifestyle behavior that can affect the quality of life and the prevalence of non-communicable diseases (NCDs) including dementia (16). For example, it has been demonstrated the beneficial effects of healthy dietary patterns such as the Mediterranean diet, or the Dietary Approach to Stop Hypertension (DASH) diet on cognition (17, 18). The role of individual nutrients including vitamins and minerals in cognitive function is also an area getting increasing attention in recent years. With regard to the association between dietary minerals and cognitive function, the results of human studies are inconclusive.

In the current study, we used data from SHNS 2018 including 835 participants and assessed the relationship between dietary intake of manganese, iron, copper, zinc, and magnesium and cognitive performance. Firstly, we found the positive association between higher copper dietary intake and lower rates of low MMSE score among the adults above 60 years old. These results are consistent with a previous perspective cohort study in Chinese elderly (aged over or equal to 55 years old) showing that the cognitive decline decreased significantly with the increment of dietary copper intake (19). In that study, the decrease reached a plateau when the intake of dietary copper exceeded about 1.3 mg/day. Our results also observed the threshold effects of copper intake on cognitive function. Moreover, the median dietary intake of copper in our current study is around 1.51 mg/day, which is much higher than RNI of 0.8 mg/day. The sufficient dietary copper intake may be due to participants’ traditional “Southern River -style diet,” which include large amounts of grains, legumes and marine fish (20). Although animal studies have demonstrated that brain copper overload is involved in the pathogenesis of neurodegeneration by enhancing oxidative damage and neuroinflammation, copper is also a co-factor of key enzymes and a signaling and regulatory molecule for brain development and function (21). Our study found the that the dietary copper intake was lower in elderly adults over 60 years old, compared to adults under 60 years old. Even the dietary copper intake meets RNI, higher dietary intake of copper may prevent cognitive decline in elderly population over 60 years old.

Secondly, we found the trend of relationship between higher magnesium intake and lower rates of low MMSE score. Our findings are consistent with another cross-sectional study from China observing the inverse association between high magnesium concentration and the incidence of mild cognitive impairment (MCI) in participants over the age of 60 (22). The beneficial effects of magnesium may be due to its ability to suppress inflammation by modulating inflammatory mediators such as interleukin-*α*, tumor necrosis factor-α and nitric oxide, and inhibit the excessive production of amyloid *β*-protein that are involved in the process of neurodegeneration (23, 24). In addition, magnesium is also important for optimal nerve transmission and neuromuscular coordination (25). It is worth mentioning that the median dietary intake of magnesium in our present study is 251.57 mg/day, which is below RNI for individuals in China. It is necessary for elderly adults over 60 years old to eat magnesium rich food to meet RNI and prevent cognitive decline.

In the present study, we found that elderly ate fewer calories with higher dietary intake of copper and magnesium than young adults. Other studies also supported the findings that the elderly had increased demand for healthy food rich in nutrients and bioactive compounds as they were more concerned about health (26). One study found that there was a remarkably energy excess derived from fat in younger group, if compared with the recommended allowances (27). Another study found that older outpatients reported higher frequency of consumption of fresh fruit and vegetables, and lower daily consumption of sweet high-fat foods among obese and overweight outpatients (28).

Regarding other minerals including iron, manganese and zinc, we did not observe the association of the dietary intake and cognitive function. Previous studies also showed no significant association of the dietary intake of zinc, selenium and manganese with cognition (29, 30). However, some other studies observed the relationship between high iron intake and poor cognition (31, 32). The different results could be explained by the assessment methods for dietary mineral intake and cognitive function. Thus studies with larger dataset and more participants are needed to further examine the association between the dietary mineral intake and cognitive outcomes. Moreover, we found that the median dietary selenium intake was lower than RNI, while the median dietary zinc intake just met the RNI of female and was lower than the RNI of male. We also found that individuals over 60 years old had lower dietary zinc and selenium intake compared to individuals under 60 years old. Even our study shows no association between zinc or selenium intake and cognitive function, adequate zinc and selenium intake play important roles in health maintenance in elderly population. Zinc deficiency is reported to affect immune function, cognitive ability, taste and many other aspects of health problems (32). Selenium is considered as longevity indicator and inadequate selenium status might accelerate the aging process or increase risk of various diseases including immunity dysfunction, neurodegeneration and cancers (33). Our study suggested local residents in Shanghai to eat a diet rich in zinc and selenium, especially for the elderly over 60 years old.

We recognized several limitations of the current study. First, our study is a cross-sectional study with a number of unmeasured confounders that may affect the dietary intake. So it is difficult to establish a causal relationship between dietary intake and cognitive performance. It is possible that other factors such as health status, emotional stress and medications may temporarily affect the cognitive test results, and thus further prospective long term cohort studies are needed to confirm the association. Second, our study did not provide a good indication of metabolism and absorption of the minerals since data on serum levels of minerals were not included in the analysis. Thirdly, our dietary intake data were derived from the 24-h dietary recall, which may be subject to recall bias and subjective dietary assessment. In addition, cognitive dysfunction may be associated with complex metal dis-regulation in the brain, which cannot be assessed through diet alone. Thus the underlying mechanisms of action of these minerals in the brain needs deeper exploration.

In conclusion, our findings suggest that dietary intake of copper and magnesium may have a protective effect on cognitive performance. It is necessary for Chinese individuals over 60 years old to get recommended amounts of copper and magnesium from diet or supplements. However, our current study is a preliminary screening of the community population. Thus further high quality prospective cohort studies to characterize the stage of dementia are necessary to confirm these findings and reveal the underlying mechanisms.

## Data Availability

The raw data supporting the conclusions of this article will be made available by the authors, without undue reservation.
