# Supplementary material for: The associations of dietary manganese, iron, copper, zinc, selenium and magnesium with cognitive outcomes in Chinese adults: a cross sectional study in Shanghai
Source: Front Nutr. 2024 Dec 4;11:1424614. doi: 10.3389/fnut.2024.1424614 (PMC11652161; doi:10.3389/fnut.2024.1424614)

Figure S1 calculated the the contribution of various foods to copper and magnesium intake. The results showed that grains were the primary contributor to the copper and magnesium intake (32%), followed by vegetables (11.9%), legumes (10.7%), sea food (10.4%), meats and poultry (8.5%), fruits (5%), fast food (4.3%), cookies and snacks (3.3%), eggs (2.6%), fungi and alage (2.4%), and nuts (2.2%).

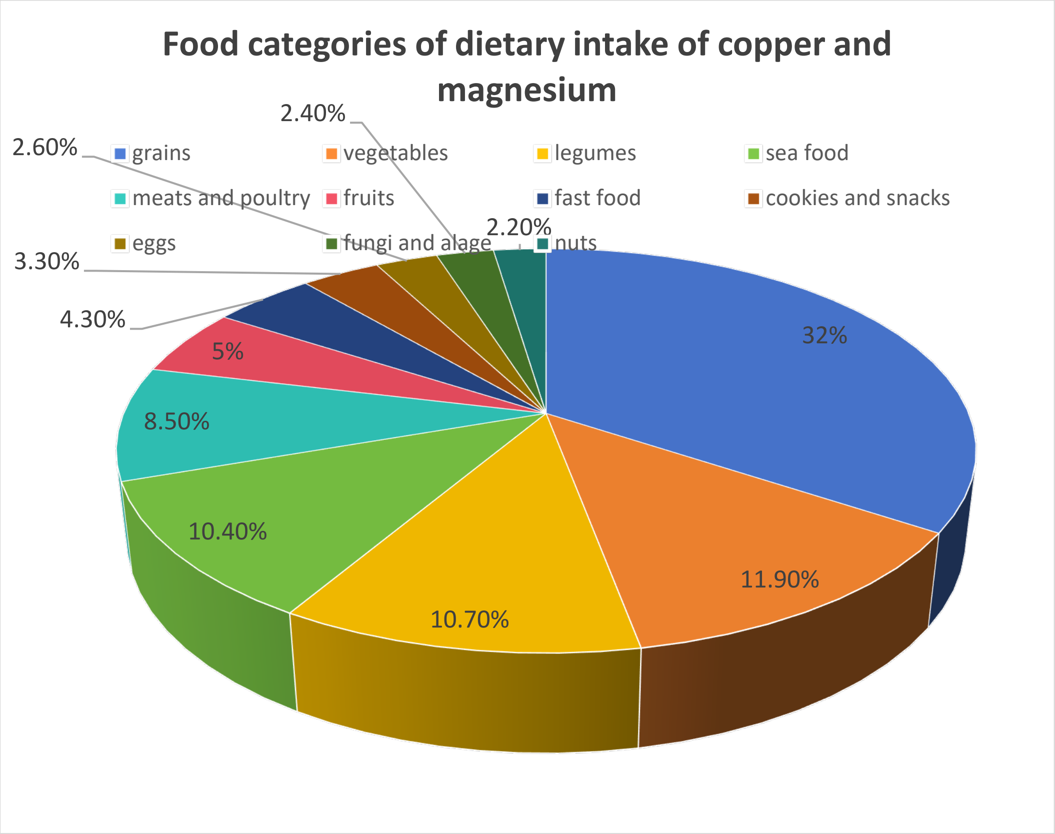

Figure S2 performed a correlation analysis of dietary mineral intake, showing that all minerals have a significant correlation.

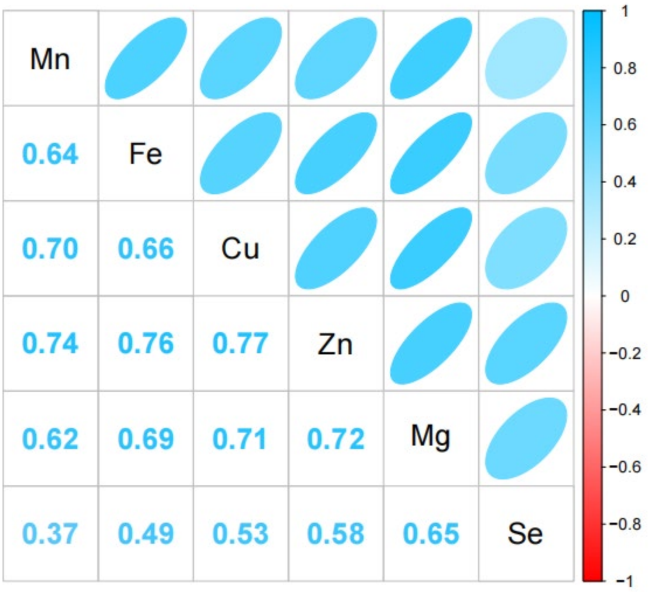

Supplement: Supplementary file 4 [file Image_1.pdf]
